# Supplementary material for: Long noncoding RNA related to periodontitis interacts with miR-182 to upregulate osteogenic differentiation in periodontal mesenchymal stem cells of periodontitis patients
Source: Cell Death Dis. 2016 Aug 11;7(8):e2327–. doi: 10.1038/cddis.2016.125 (PMC5108307; doi:10.1038/cddis.2016.125)
Supplement: Supplementary Information [file cddis2016125x1.docx]

**SUPPLEMENTAL DATA**

**MATERIALS AND METHODS**

**Cells culture**

Healthy and inflammatory tissues were obtained from the middle 1/3 of teeth roots which washed by sterile phosphate-buffered saline (PBS). These tissues were cutted into small pieces and digested with type 1collagenase (0.66 mg/mL; Sigma, St Louis, MO, USA) for 20 minutes. Then the cell suspensions were filtered and cultured in aminimum essential medium (a-MEM; Gibco BRL, Gaithersburg, MD, USA) supplemented with 10% fetal bovine serum (FBS),

0.292 mg/mL of glutamine (Invitrogen, Carlsbad, CA, USA), 100 U/mL of penicillin, and 100 mg/mL of streptomycin (Gibco BRL) at 37℃ in a humidified atmosphere of 5% CO2 and 95% air. After about two weeks, the cells from healthy and inflammatory tissues were digested by trypsin and single cell-derived colony cultures were obtained using the limiting dilution technique.

Multiple colony-derived PDLSCs were used in this study after two to four passages. For each experiment, the same passage of PDLSCs was used.

**TNF-α** **and IL-1β treatment**

For TNF-α and IL-1β treatment, we treated hPDLSCs with TNF-α (10 ng/mL) and IL-1β (5 ng/mL) in basic medium for 7 days. Then the culture medium was changed to osteogenic medium, which still contained TNF-α (10 ng/mL) and IL-1β (5 ng/mL) and was used for hPDLSC cultures for 7 or 14 days.

**Flow cytometric analysis**

For identification of the MSC phenotype, 5 × 10^5^ cells were incubated with PE or FITC conjugated monoclonal antibodies for human CD14, CD31, CD90, CD105 (eBioscience, San Diego, CA, USA), CD146 and Stro-1 (R&D Systems, Inc., Minneapolis, MN, USA), or isotype-matched control IgGs. Cells were subjected to flow cytometric analysis using a Beckman Coulter Epics XL (Beckman Coulter, Fullerton, CA, USA).

**Colony forming unit assay(CFU)**

Single-cell suspensions contained 2× 10^3^ cells were seeded in 10-cmdiameter

culture dishes(Corning, Lowell, MA, USA) and cultured for 7 days,

The formed colonies were fixed bv 4% paraformaldehyde fixation and then were visualized with 0.1% toluidine blue. Colonies were defined as Aggregates which containing more than 50 cells and were scored under the microscope (Leica Microsystems, Heerbrugg, Switzerland). CFU efficiency was determined by the number of colonies relative to the total number of seeded cells in each plate. This experiment was repeated for three times.

**Adipogenic differentiation**

Cells ( 1×10^5^/well) were culture in 6-well plates until reached 80% confluence. Then these cells were incubated with adipogenic medium (0.5 mM

methylisobutylxanthine, 0.5 mM hydrocortisone, and 60 mM indomethacin (Sigma) for 21 days. Cells were washed with 10% fetal bovine serum (PBS) 2 times and fixed ith fixed with 4% paraformaldehyde and stained with oil red O solution. This experiment was repeated for three times.

**Cytokine expression assays**

TNF-α and IL-1β in the culture supernatant of hPDLSCs, pPDLSCs, and hPDLSCs with TNF-α and IL-1β stimulation were determined using ELISA kits (R&D Systems, USA) according to the manufacturer's protocols.

**Osteogenic differentiation**

Cells (1×10^5^/well) were cultured in 6-well plates until they reached 80% confluence. Then, these cells were incubated with osteogenic medium (100-nM dexamethasone, 50-mg/ml ascorbic acid, and 5-mM b-glycerophosphate; Sigma, St. Louis, MO, USA) for 7 or 14 days, and the media was changed every other day. The alkaline phosphatase (ALP) activity assay was performed after 7 days of incubation with osteogenic medium using an ALP kit according to the manufacturer's protocol (Jiancheng, Nanjing, China). ALP staining was performed using the 5-Bromo-4-chloro-3-indolyl phosphate (BCIP)/ Nitro Blue Tetrazolium (NBT) Alkaline Phosphatase Color Development Kit according to the manufacturer's protocol (Beyotime, Shanghai, China). Alizarin red staining was performed after 14 days of incubation with osteogenic medium. Cells were washed with 10% fetal bovine serum (in PBS) 2 times and fixed with 60% isopropanol for 1 minute. Then, the cells were rehydrated with distilled water for 3 minutes and stained with 1% Alizarin red (Sigma, St. Louis, MO, USA). To quantify the alizarin red–stained nodules, the stain was solubilized with 0.5 mL of 5% SDS in 0.5N HCl for 30 minutes at room temperature. Solubilized stain (0.15 mL) was transferred to the wells of a 6-well plate, and absorbance was measured at 405 nm. All experiments were repeated for three times.

**qPCR**

Total RNA, inclusive of the small RNA, was extacted using TRIzol reagent (Invitrogen) and converted into cDNA (SuperScript First-Strand Synthesis Kit; Invitrogen). The miR-182 RNAs were reverse transcribed using a specific RT primer (RiboBio, Guangzhou, China) according to the manufacturer's protocol. β-Actin and was used as endogenous normalization controls for mRNAs and lncRNAs. U6 was used as endogenous normalization controls for miR-182. Primer pairs for all lncRNAs and miR-182 were designed by RiboBio (Guangzhou, China). Other primer pairs are listed in Supplementary Table S1.

qPCR was performed using the QuantiTect SYBR Green PCR Kit (Toyobo, Osaka, Japan) and the Applied Biosystems 7500 Real-Time PCR Detection System. The data were analyzed using the 2^−ΔΔCt^ (Livak) relative expression method. All experiments were repeated three times.

**Western blot**

Total protein was extracted from the cells by lysis in radioimmunoprecipitation assay (RIPA) buffer (10 mM Tris–HCl, 1 mM EDTA, 1% sodium dodecyl sulfate, 1% Nonidet P-40, 1:100 proteinase inhibitor cocktail, 50 mM b-glycerophosphate, and 50 mM sodium fluoride). Cell samples were fractionated into the cytosolic and nuclear fractions using the NE-PER Nuclear and Cytoplasmic Extraction Kit (Thermo, USA) according to the manufacturer's protocols. The protein content of the lysate was determined using a protein assay kit (Beyotime) according to the manufacturer's protocol. Then, 20 mg of the cell lysate samples were separated by 10% sodium dodecyl sulfate polyacrylamide gel electrophoresis (SDS-PAGE), and the proteins were then transferred to a polyvinylidene fluoride (PVDF) membrane (Bio- Rad, Hercules, CA, USA). The membranes were blocked with 5% milk for 2 hours and then incubated with primary antibodies for one night at 4 ºC. Immune complexes were incubated with horseradish peroxidase-conjugated anti-rabbit or anti-mouse IgG antibodies (Boshide, Beijing, China). Immunodetection was performed using the Western-Light Chemiluminescent Detection System (Peiqing, Shanghai, China). All experiments were repeated three times.

Primary antibodies were purchased from the following commercial sources:, Runx2 (1:500, Abcam , Cambridge, MA,USA), β-catenin (1:800, Abcam) , FoxO1(1:1000, Cell Signaling Technology, MA,USA ), TCF-4 (1:500, Cell Signaling) β-actin(1:800,Abcam), HDAC1(1:800,Cell Signaling), anti-rabbit or anti-mouse IgG antibodies(1: 10000, Boshide, Beijing, China)

**RESULTS**

**Identification of hPDLSCs and pPDLSCs** The hPDLSCs and pPDLSCs were isolated by limiting dilution technique. Both of these two types of cells can express MSC markers (Figure S1A) and form colony-forming unit-fibroblast generated from single cells (Figure S1B). To indentify the multidifferentiation potential hPDLSCs and pPDLSCs, we found both two types of cells could form mineralized nodules (FigureS1C) or lipid droplets (Figure S1D) under osteogenic or adipogenic induction. All these strongly demonstrate that hPDLSCs and pPDLSCs are mesenchymal stem cells.

**Inflammatory microenvironments lead to osteogenic differentiation deficiency in later passage pPDLSCs.** To indentify the effects of inflammatory microenvironments on the osteogenesis of hPDLSCs, all cells used in this part were 7 passages cells. hPDLSCs, pPDLSCs and hPDLSCs, which were treated with TNF-α and IL-1β (h+ cytokines), were cultured in osteogenic medium for 7 or 14d. Then the Alizarin red staining, ALP staining and ALP activity assays revealed that the osteogenesis was impaired in pPDLSCs and h+ cytokines (Figure S2A-D)**.** qPCR also showed mRNA levels of osteogenic genes including Runx2, ALP and Col1 were significantly decreased in pPDLSCs and h+ cytokines, compared with hPDLSCs. (Figure S 2E) Besides, we also measured TNF-α and IL-1β concentration in conditioned medium through ELISA and found pPDLSCs and h+ cytokines secreted more cytokines than hPDLSCs. (Figure S 2F)

**Supplementary Table S1**

**Primers of lncRNAs for qPCR**

| Primer name | Sequence (5’ to 3’) |
| --- | --- |
| ENST00000445235 | F: 5’- TGGTATCAGAAGGTGGGGACTT-3’  R: 5’-AGAAGGCTGTCATTTTGCTGTG-3’ |
| ENST00000512720 | R: 5’-AGAAGGCTGTCATTTTGCTGTG -3’  R: 5’-CTGTCCCCAAATCACCCTTC -3’ |
| ENST00000446358 | F: 5’-CATGTTTGTCTGAACTTCGTCTTC-3’  R: 5’-GTTATAATTTGGAGGGCAACTAGG-3’ |
| ENST00000537192 | F: 5’-AGGTGACTGCCTTCCTTTCATC -3’  R: 5’-GCCACACATTGACCATTCCTT -3’ |
| ENST00000431757 | F: 5’-AGCTGTTGGAGTAGCACTGTATTTG -3’  R: 5’- CCTACCAGCCTCTTCCTGTAATGT-3’ |
| ENST00000523380 | F: 5’-TGGAGAAGTGGGGAAGGTG -3’  R: 5’-GGGAGGAGCATCAGGTTGTTT -3’ |
| ENST00000567058 | F: 5’-GTCTCTGGCAACCTCACAACC -3’  R: 5’-GAGCCCAGGAGTTCAAGATTACA-3’ |

**Primers of mRNAs for qPCR**

| Primer name | Sequence (5’ to 3’) |
| --- | --- |
| β-actin | F: 5’- TGGCACCCAGCACAATGAA -3’  R: 5’- CTAAGTCATAGTCCGCCTAGAAGCA -3’ |
| Runx2 | F: 5’- CCCGTGGCCTTCAAGGT -3’  R: 5’- CGTTACCCGCCATGACAGTA -3’ |
| ALP | F: 5’- GGACCA TTCCCACGTCTTCAC -3’  R: 5’- CCTTGTAGCCAGGCCCATTG -3’ |
| Col1 | F: 5’- CCAGAAGAACTGGTACATCAGCAA -3’  R: 5’- CGCCATACTCGAACTGGAATC -3’ |
| FoxO1 | F: 5’- ACGAGTGGATGGTGAAGAGC -3’  R: 5’- TGCTGTGAAGGGACAGTTG -3’ |
| cyclin D1 | F: 5’- TGATGCTGGGCACTTCATCTG -3’  R: 5’- TCCAATCATCCCGAATGAGAGTC -3’ |
| c-myc | F: 5’- GAATGTCAAGAGGCGAACACA -3’  R: 5’- CGTCGTTTCGCGAACAAG -3’ |
| IKKα | F: 5'-GTGAACATCCTCTGACATGTGTGGT-3'  R: 5'-GCAACACAAGGAGGCTGGGCT-3' |

**Primers of the immunoprecipitated DNA for ChIP**

| Primer name | Sequence (5’ to 3’) |
| --- | --- |
| Primer 1: control  (regions without binding sites of P65/c-Rel) | F: 5’-TCCTTTCGGGCAGAGGTGTC-3’  R: 5’-GGTGGAGTGAGATGGGATGATGG-3’ |
| Primer 2: binding regions  (regions with binding sites of P65/c-Rel) | F: 5’-CTTGCTAAAGGCTTGAGATGC-3’  R: 5’-CTCCTGACCACAGAACCCAC-3’ |

**Supplementary Table S2**

**Comparison between the array data and qPCR data**

|  | **array data** | | **qPCR data** | |
| --- | --- | --- | --- | --- |
| **lncRNA name** | **Fold change** | **P-value** | **Fold change** | **P-value** |
| ENST00000523380 | 7.5225 ( up) | 0.0009 | 7.6859 (up) | 0.0057 |
| ENST00000567058 | 5.5944 (up) | 0.0012 | 3.7477 (up) | 0.0206 |
| ENST00000445235 | 31.8226 (down) | 0.0409 | 29.218 (down) | 4.3585E-06 |
| ENST00000512720 | 8.7883 (down) | 0.0312 | 8.7716 (down) | 0.0416 |
| ENST00000446358  (lncRNA POIR) | 5.8380 (down) | 0. 0014 | 5.5034 (down) | 0.0001 |
| ENST00000537192 | 5.0696 (down) | 0.0128 | 4.8037 (down) | 0.0007 |
| ENST00000431757 | 5.0409 (down) | 0.0101 | 2.9588 (down) | 0.0661 |

*Up: compared with hPDLSCs, lncRNA in pPDLSCs has been up regulated.

Down: compared with hPDLSCs, lncRNA in pPDLSCs has been down regulated.

**Supplementary Table S3**

**Grouping situation of in vivo transplantation**

| Objective | Grouping situation |
| --- | --- |
| Detect the osteogenic effect of lncRNA-POIR in pPDLSCs (Results could be found in **Figure 2**.) | pPDLSCs, pPDLSCs with shlncRNA negative control, pPDLSCs with shlncRNA, pPDLSC with lncRNA overexpression negative control, pPDLSC with lncRNA overexpression, |
| Detect the osteogenic effect of lncRNA-POIR in hPDLSCs (Results could be found in **Figure 3**.) | hPDLSCs, hPDLSCs with shlncRNA negative control, hPDLSC with lncRNA overexpression |
| Detect the osteogenic effect of FoxO1 in pPDLSCs (Results could be found in **Figure 6**.) | hPDLSCs, pPDLSCs, pPDLSCs with siNC, pPDLSCs with siFoxO1 |

**Supplementary Figure 1** Identification of hPDLSCs and pPDLSCs (A) Mesenchymal stem cell phenotype examination by flow cytometric analysis. (B) Representative images of colonies formed by hPDLSCs and pPDLSCs at low seeding density after 14 days of culture(magnification: 100×). (C) Osteogenic differentiation was determined by Alizarin Red staining after 14 days osteogenic induction. (D) Adipogenic differentiation was evaluated by Oil Red O staining after 21 days adipogenic induction. The scale bar in the micrographs represents 100 nm.

**Supplementary Figure 2** The osteogenic differentiation deficiency in later passage pPDLSCs. (A, C) Osteogenic differentiation of hPDLSCs, pPDLSCs and h+ cytokines were determined by Alizarin Red staining after 14 days osteogenic induction. (B, D) Osteogenic differentiation of hPDLSCs, pPDLSCs and h+ cytokines were determined by ALP staining after 7 days osteogenic induction. (E) Runx2, ALP and Col1expressions were measured by qPCR in hPDLSCs, pPDLSCs and h+ cytokines after 7 days osteogenic induction. (F) The concentration of secreted cytokines in the hPDLSCs, pPDLSCs and h+ cytokines culture medium were determined by ELISA. All experiments were repeated three times. Relative expressions of mRNAs were normalized by β-action in qPCR. Data represent mean ± S.D. *P < 0.05, **P < 0.01, NS, not significant. The scale bar in the micrographs represents 100 nm. Abbreviations: h+ cytokines: hPDLSCs treated with TNF-α and IL-1β; OD, optical density.

**Supplementary Figure 3** Differentially expressed mRNAs between

hPDLSCs and pPDLSCs. (A) Heat map of differentially expressed mRNAs (the top 10 in up-regulated mRNAs and the top 10 in down-regulated mRNAs) between hPDLSCs and pPDLSCs. (B, C) Go analysis covers the three domains of biological process, cellular component and molecular function. C: up-regulated GO terms in pPDLSCs; D: down-regulated GO terms in pPDLSCs (D, E) Pathway analysis showed that the most significant pathways consisted of focal adhesion, ECM-receptor interaction and bacterial invasion of epithelial cells, Long-term depression, Circadian entrainment, HIF-1 signaling pathway. C: up-regulated pathways in pPDLSCs; D: down-regulated pathways in pPDLSCs. All experiments were repeated three times. A total of 6 samples (3 pPDLSCs and 3 hPDLSCs from 6 individuals) are tested in lncRNA and mRNA profiling.

**Supplementary Figure 4** The negative effects of miR-182 on osteogenic differentiation. The effects of anti-miR-182 on osteogenesis of pPDLSCs were determined by qPCR (A), western blot (B) and Alizarin red staining (C, D) 7 or 14 days after osteogenic induction. All experiments were repeated three times. Relative expressions of mRNAs were normalized by β-action in qPCR. Data represent mean ± S.D. *P< 0.05, **P < 0.01, NS, not significant. Abbreviations: Con, Control; anti-miR-NC, siPORT reagent alone; anti-miR182, miR182 inhibitor; OD, optical density.
