# Supplementary figures and images for: Long noncoding RNA related to periodontitis interacts with miR-182 to upregulate osteogenic differentiation in periodontal mesenchymal stem cells of periodontitis patients
Source: Cell Death Dis. 2016 Aug 11;7(8):e2327–. doi: 10.1038/cddis.2016.125 (PMC5108307; doi:10.1038/cddis.2016.125)

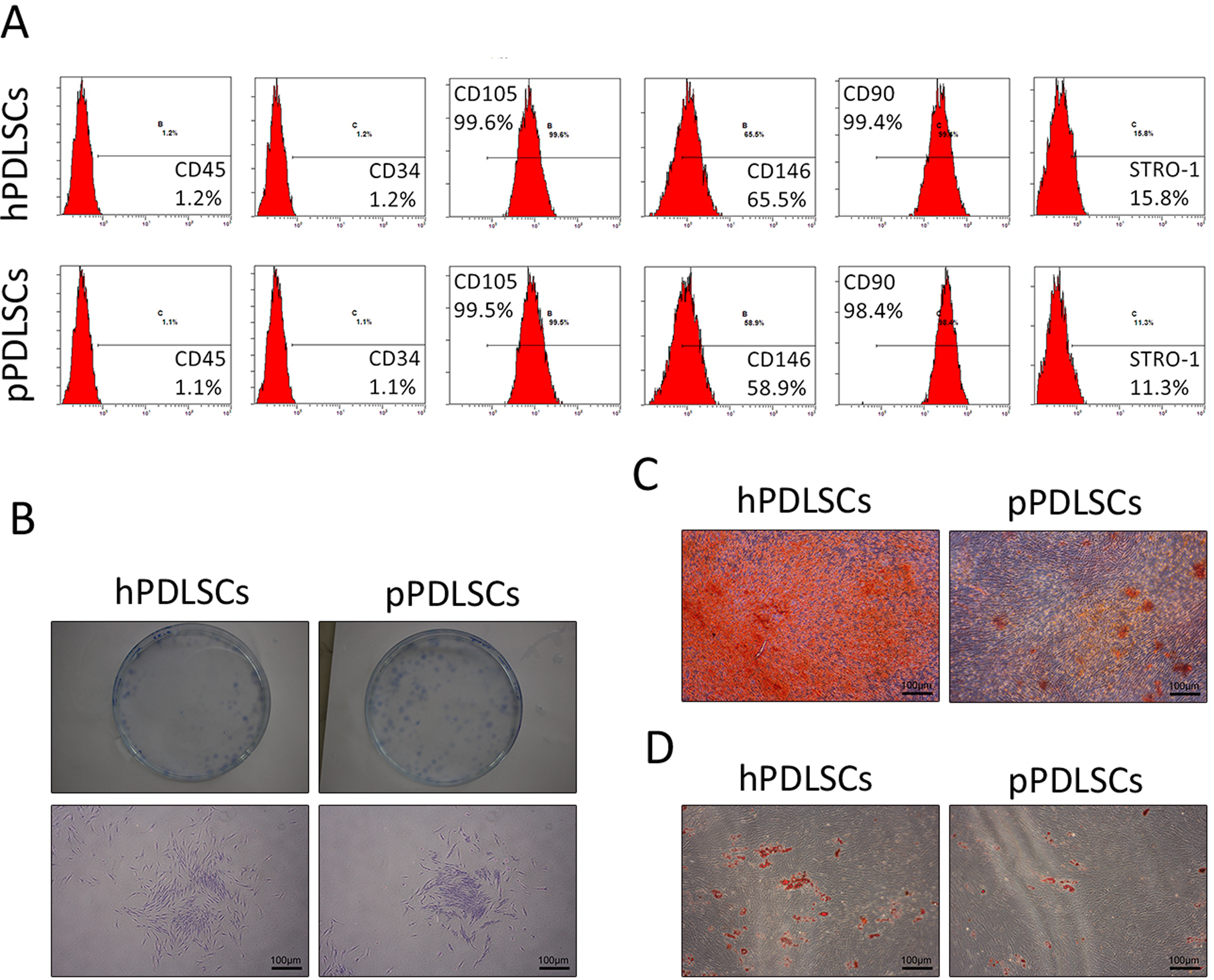

Supplement: Supplementary Figure 1 [file cddis2016125x2.tif]

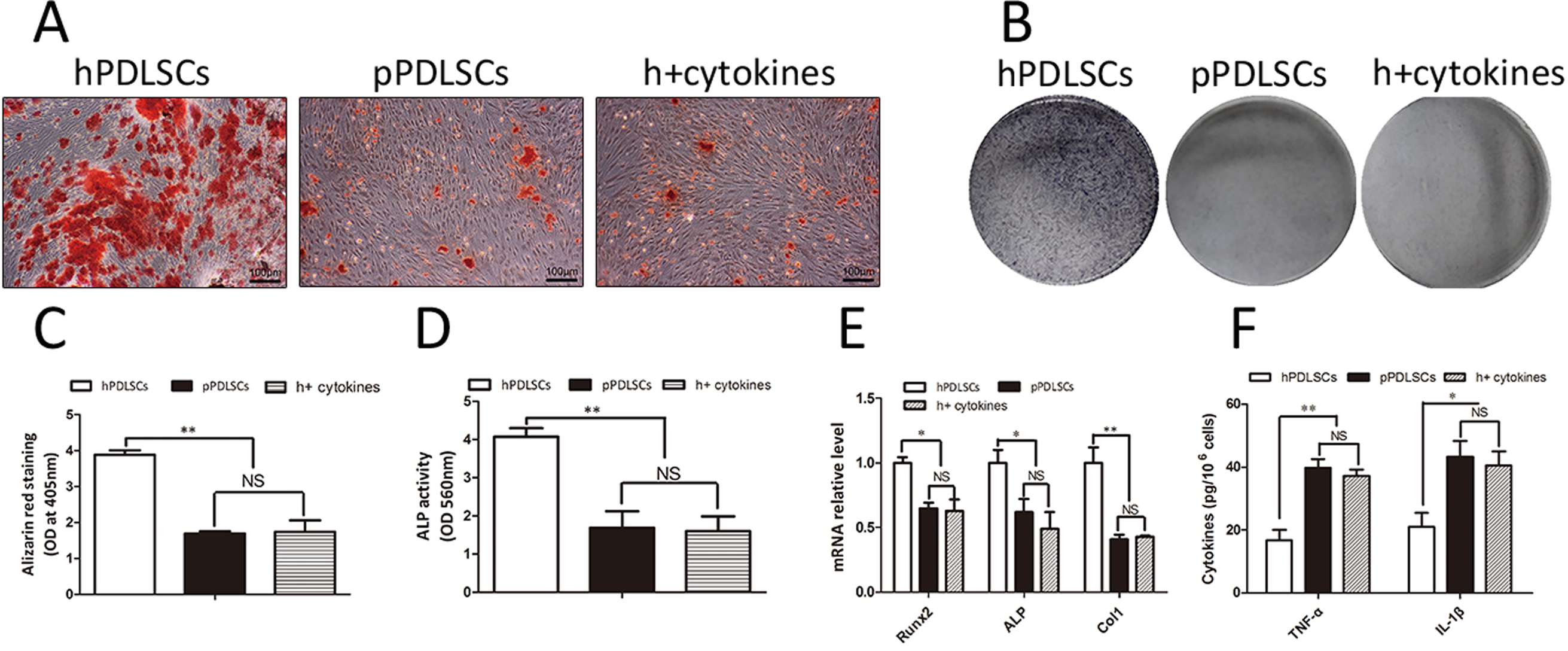

Supplement: Supplementary Figure 2 [file cddis2016125x3.tif]

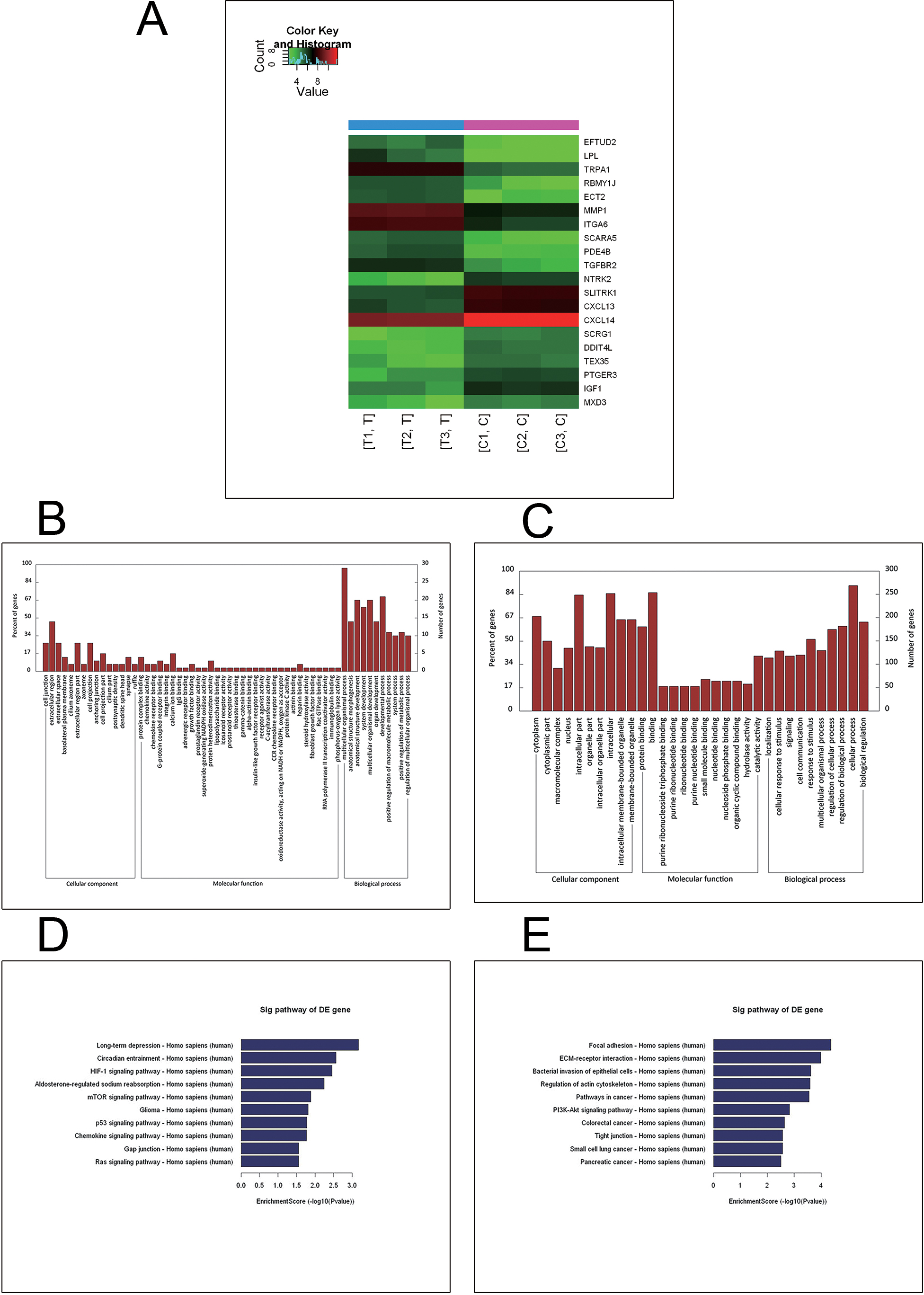

Supplement: Supplementary Figure 3 [file cddis2016125x4.tif]

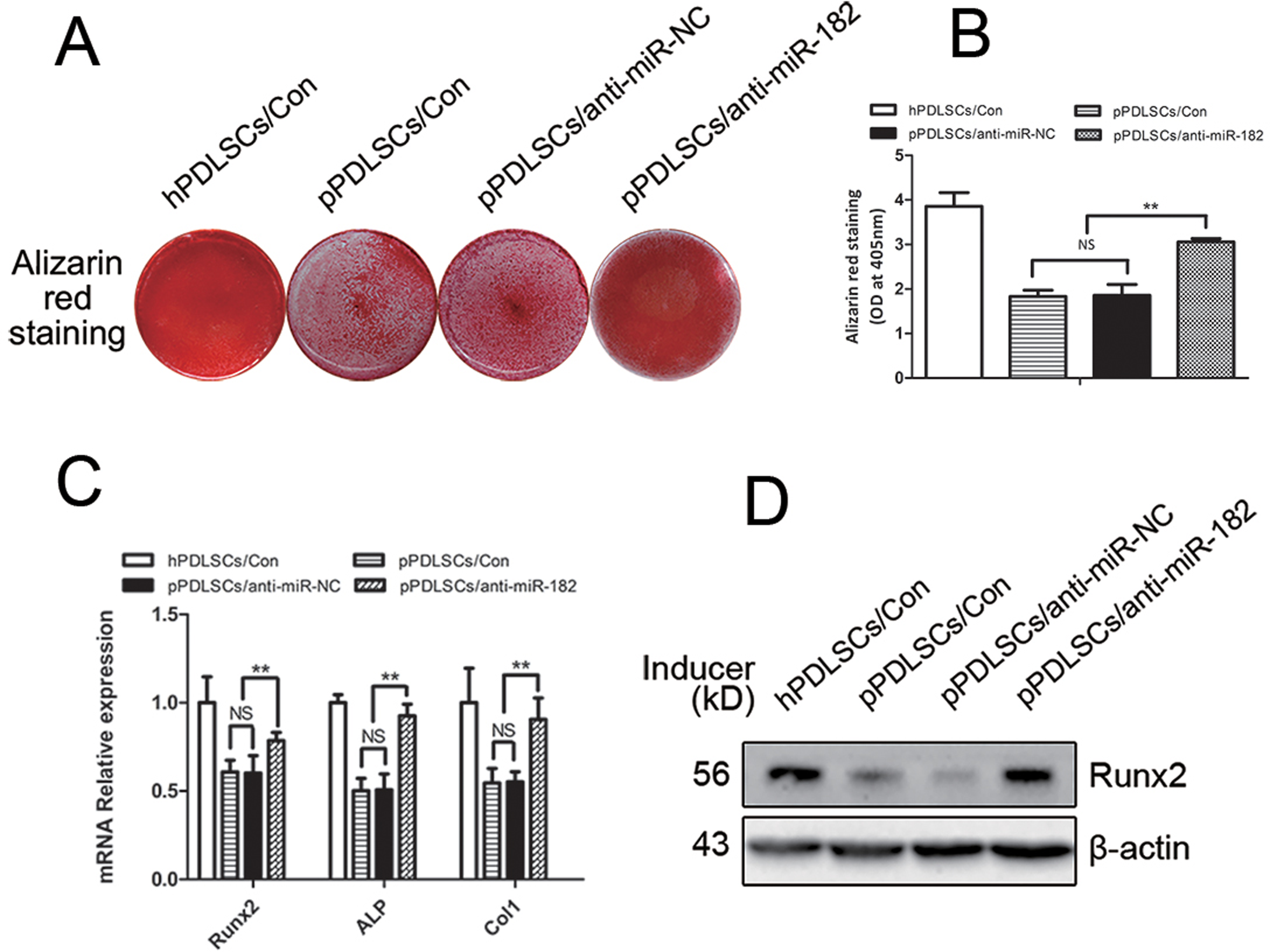

Supplement: Supplementary Figure 4 [file cddis2016125x5.tif]
